# Antidepressants that increase mitochondrial energetics may elevate risk of treatment-emergent mania

**DOI:** 10.1038/s41380-022-01888-x

**Published:** 2022-12-13

**Authors:** Manuel Gardea-Resendez, Brandon J. Coombes, Marin Veldic, Susannah J. Tye, Francisco Romo-Nava, Aysegul Ozerdem, Miguel L. Prieto, Alfredo Cuellar-Barboza, Nicolas A. Nunez, Balwinder Singh, Richard S. Pendegraft, Alessandro Miola, Susan L. McElroy, Joanna M. Biernacka, Eva Morava, Tamas Kozicz, Mark A. Frye

**Affiliations:** 1 Department of Psychiatry & Psychology, Mayo Clinic, Rochester, MN USA; 2 Department of Psychiatry, Universidad Autónoma de Nuevo León, Monterrey, Mexico; 3 Department of Quantitative Health Sciences, Mayo Clinic, Rochester, MN USA; 4 Queensland Brain Institute, The University of Queensland, St. Lucia, QLD Australia; 5 Lindner Center of HOPE /Department of Psychiatry and Behavioral Neurosciences, University of Cincinnati College of Medicine, Mason, OH USA; 6 Department of Psychiatry, Facultad de Medicina, Universidad de los Andes, Santiago, Chile; 7 Mental Health Service, Clínica Universidad de los Andes, Santiago, Chile; 8 Department of Neuroscience (DNS), University of Padova, Padua, Italy; 9 Department of Clinical Genomics, Mayo Clinic, Rochester, MN USA; 10 Department of Anatomy, University of Pecs, Medical School, Pecs, Hungary; 11 Center for Individualized Medicine, Mayo Clinic, Rochester, MN USA

**Keywords:** Bipolar disorder, Cell biology

## Abstract

Preclinical evidence suggests that antidepressants (ADs) may differentially influence mitochondrial energetics. This study was conducted to investigate the relationship between mitochondrial function and illness vulnerability in bipolar disorder (BD), specifically risk of treatment-emergent mania (TEM). Participants with BD already clinically phenotyped as TEM+ (*n* = 176) or TEM− (*n* = 516) were further classified whether the TEM associated AD, based on preclinical studies, increased (Mito+, *n* = 600) or decreased (Mito−, *n* = 289) mitochondrial electron transport chain (ETC) activity. Comparison of TEM+ rates between Mito+ and Mito− ADs was performed using generalized estimating equations to account for participants exposed to multiple ADs while adjusting for sex, age at time of enrollment into the biobank and BD type (BD-I/schizoaffective vs. BD-II). A total of 692 subjects (62.7% female, 91.4% White, mean age 43.0 ± 14.0 years) including 176 cases (25.3%) of TEM+ and 516 cases (74.7%) of TEM- with previous exposure to Mito+ and/or Mito- antidepressants were identified. Adjusting for age, sex and BD subtype, TEM+ was more frequent with antidepressants that increased (24.7%), versus decreased (13.5%) mitochondrial energetics (OR = 2.21; *p* = 0.000009). Our preliminary retrospective data suggests there may be merit in reconceptualizing AD classification, not solely based on monoaminergic conventional drug mechanism of action, but additionally based on mitochondrial energetics. Future prospective clinical studies on specific antidepressants and mitochondrial activity are encouraged. Recognizing pharmacogenomic investigation of drug response may extend or overlap to genomics of disease risk, future studies should investigate potential interactions between mitochondrial mechanisms of disease risk and drug response.

## Introduction

As the pharmacopoeia for major depressive episodes in bipolar disorder (BD) is markedly underdeveloped, antidepressants are invariably used with little evidence base. This clinical practice is of significant consequence as antidepressant prescriptions for BD in the USA have more than doubled in the last two decades from 17.9% to 40.9% [1]. In addition to relatively high rates of treatment non-response, antidepressants have the potential to increase the likelihood of a switch process, invariably defined as antidepressant-induced mania (AIM) [2], treatment-emergent mania (TEM+) [3], and/or cycle acceleration [4]. The increased energy expenditure of mania associated with impulsivity, poor judgment, psychosis, and/or loss of insight can drive high risk behaviors often resulting in hospitalization or incarceration; further, the aftermath of mania can have enduring negative impact on quality of life [5–7].

A random effects meta-analysis of 35 clinical trials of bipolar depressed patients reported a switch rate of 12.5% with and 7.5% without antidepressant use [8]. A Swedish registry study identified that patients with BD treated with antidepressant (AD) monotherapy, in comparison to AD with concurrent mood stabilization, were at significant increased risk of treatment-emergent mania (TEM+), most notably, during the first 3 months of treatment (hazard ratio=2.83, 95% CI = 1.12, 7.19) [9]. The clinical factors most associated with TEM+ include younger age, female sex, mixed symptoms, and type I BD [6]. As there is increasing interest is developing a cumulative risk model of TEM+ based on clinical and biological markers, when not use an antidepressant is a focused area of biomarker development with great potential to impact practice by primary or secondary prevention of mania [10, 11].

It has long been recognized that the neurobiology of BD is driven, in part, by mitochondrial dysfunction as exemplified by reduced expression of electron transport chain (ETC) genes in frontal cortex and hippocampus [12, 13]. The resulting impaired oxidative phosphorylation with a shift towards glycolysis and overall decreased adenosine-5’-triphosphate (ATP) production in response to energy demands has been proposed to be one of the main drivers of BD pathophysiology [14, 15]. Suboptimal mitochondrial function (SMF) in BD has been operationalized at several critical time points of illness vulnerability including early brain development resulting in structural and/or functional change in plasticity, genetic risk before illness, relapse risk into mania, psychosis, or depression in established disease, and relapse based on non-specific symptoms characteristic to mitochondrial disorders (i.e., fatigue, circadian rhythm disturbance) [16–19].

Targeting antidepressants and mitochondrial function is further justified based on our preclinical rodent model of ACTH-driven, imipramine treatment-resistant depression whereby electrode implantations to the nucleus accumbens elicited mania-like behavior in a subset of animals (30%). This behavioral phenotype was associated with increased mitochondrial respiration, specifically an increased state 3/state 4 respiration control ratio (RCR), suggestive of increased respiratory efficiency [20]. This finding was not driven exclusively by imipramine, the tricyclic antidepressant first shown in controlled evaluations to be associated with a high incidence of mania [31], but more likely an interaction between imipramine and an acute inflammatory response associated with DBS electrode placement in nucleus accumbens [47].

Mechanistically, antidepressants have been shown to differentially impact ETC complex activity [21–23]. While the specific mechanism of antidepressant associated increase of ETC activity is not fully understood, animal models suggest an upregulation of mitochondrial activity, including cellular respiration, occurring during acute antidepressant treatment, followed by decreased or unchanged activity in chronic treatment (≥28 days) [24]. The purpose of our study was to assess whether antidepressants that increase mitochondrial activity are associated with higher rates of TEM+.

## Methods and materials

A subset of participants from the Mayo Clinic Bipolar Disorder Biobank with known history of antidepressant exposure and clinical outcome measure were included in this study [25]. The Biobank sample consists of patients aged 18 through 80 years of age at time of enrollment with a type I or II bipolar disorder or schizoaffective bipolar disorder as confirmed by structured interview. Participants completed a questionnaire focused on demographics, illness variables and environmental influences and provided a blood sample [26]. Exclusion criteria included active psychosis or active suicidal ideation. Recruitment sites for the Biobank included Mayo Clinic, Lindner Center of HOPE/University of Cincinnati, University of Minnesota, Universidad Autónoma de Nuevo Leon (Mexico) and Universidad de los Andes (Chile). Each of the study sites received approval by their institutional review and every participant provided written informed consent for inclusion. Further details on study design and phenotyping are reviewed extensively in earlier work [25].

Through the Bipolar Biobank Clinical Questionnaire (BiB-CQ), research clinicians assessed and documented comorbid conditions and psychotropics used across lifetime, including antidepressants, as well as history of TEM+ while on each medication [6]. Based on an earlier meta-analytic work, which emphasized the importance of standardizing a narrow phenotype [27], TEM+ was defined as a manic/hypomanic episode by DSM criteria occurring within 60 days of starting or increasing an antidepressant dose [6, 28]. TEM− controls were characterized as ≥60-day exposure to an antidepressant with no associated manic/hypomanic episode.

**Fig. 1:**
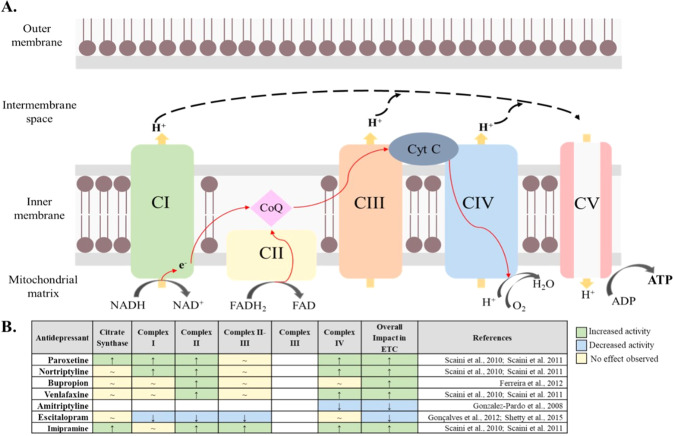
Global effect of antidepressants on mitochondrial respiratory chain complexes. **A** Schematic overview of the mitochondrial electron transport chain (ETC), a cluster of protein complexes and electron transporters in the inner mitochondrial membrane that generate ATP. The electrons generated during the oxidation of NADH and FADH_2_, in complexes I and II, respectively, are transported through coenzyme Q (CoQ), complex III (CIII), cytochrome C (Cyt C) and complex IV (CIV or COX). As electrons are transferred through the chain, energy is released to pump protons (H+), generating an electrochemical gradient across the membrane. Finally, in complex V (CV), also known as ATP synthase, the electrochemical gradient is used to catalyze the production of ATP from ADP. **B** Summary of the global effect of the investigated antidepressants and their effect on each complex of the ETC [22] based on preclinical studies [55–59]: A green box with an upward arrow indicates an increase in the activity of the specific complex after exposure to an antidepressant, a blue box with a downward arrow indicates a decrease in the activity of the specific complex after exposure to an antidepressant and a yellow box with a “~” sign indicates no effect observed. Abbreviations: CI Complex I, NADH:ubiquinone oxidoreductase, NADH reduced nictotinamide adenine dinucleotide, NAD^**+**^ nictotinamide adenine dinucleotide, CII Complex II, succinate-coenzyme Q reductase, CoQ coenzyme Q, FADH reduced flavin adenine dinucleotide, FAD flavin adenine dinucleotide, CIII Complex III, coenzyme Q – cytochrome c reductase, Cyt C cytochrome C, CIV Complex IV, cytochrome C oxidase, CV Complex V, ATP synthase, ADP adenosine diphosphate, ATP adenosine triphosphate.

Emmerzaal et al. 2021 [22], assessed the impact of psychotropic drugs on each complex of the ETC (Fig. 1) including state 3 (ADP stimulated respiration) and state 4 respiration (non-ADP stimulated), citrate synthase activity (first step of the Krebs cycle and proxy of mitochondrial mass) and malate dehydrogenase (final step of the Krebs cycle). Based on this recent preclinical review bupropion, nortriptyline, paroxetine, and venlafaxine were identified as antidepressants that increased mitochondrial function (Mito+), while amitriptyline and escitalopram as antidepressants that decreased mitochondrial function (Mito-). In this line [21], Table 1 reflects the distribution of biobank antidepressant drug exposure and clinical outcome (TEM+ vs TEM−).

**Table 1:** Participants with history of exposure to the assessed antidepressants.

**Mito+ antidepressants**	***N***	**TEM+ (*n*** = 148)	**TEM− (*n*** = 452)
Bupropion	400	68 (17.0%)	332 (83.0%)
Nortriptyline	29	2 (6.9%)	27 (93.1%)
Paroxetine	227	49 (21.6%)	178 (78.4%)
Venlafaxine	219	45 (20.6%)	174 (78.4%)
**Mito− antidepressants**		**(*n*** = 39)	**(*n*** = 250)
Amitriptyline	61	5 (8.2%)	56 (91.8%)
Escitalopram	250	35 (14.0%)	215 (86.0%)

We first assessed whether rates of TEM+ were different with respect to potential confounders such as sex, age, race, BD type, BD illness (e.g., psychosis) and psychiatric comorbidities (Table 2). We used chi-square tests and two-sample *t*-tests to formally assess these differences for categorical and continuous variables, respectively, when large differences were observed. To assess our primary aim of whether TEM+ rates differ between Mito+ and Mito- antidepressants, we compared the rate of TEM+ between Mito+ and Mito− using generalized estimating equations (using a logit link and symmetric correlation structure) to account for patients that took both Mito+ and Mito− Ads during the course of treatment. To account for the potential confounders of TEM+ rates and based on previous clinical studies, we adjusted this analysis for sex, age at time of enrollment into the biobank and BD type (BD-I/schizoaffective vs. BD-II). [6, 29, 30] As the analysis was conducted using data from retrospective assessment of TEM+, it was out of the scope of this analysis to adjust for other factors that may dynamically influence mitochondrial health, such as lifestyle factors, childhood trauma, chronic stress, exercise, and dietary habits.

**Table 2:** Demographic variables and bipolar disorder subtype in participants treated with antidepressants that increase versus decrease mitochondrial activity.

	Antidepressants that ↑ mitochondrial activity (Mito+)			Antidepressants that ↓ mitochondrial activity (Mito−)	
Variable	TEM+ (*n* = 148)	TEM− (*n* = 452)	TEM+ (*n* = 39)		TEM− (*n* = 250)
Sex (female), *n* (%)	96 (64.9%)	278 (61.5%)	28 (71.8%)		173 (69.2%)
Age, mean (SD)	41.1 ± 13.6	44.7 ± 13.7	37.9 ± 14.3		43.1 ± 13.8
Race (White), *n* (%)	131 (89.1%)	413 (92.2%)	36 (92.3%)		237 (95.2%)
Ethnicity (Hispanic), *n* (%)	13 (9.3%)	22 (4.9%)	4 (10.8%)		14 (5.6%)
SCID diagnosis, *n* (%)					
Type I BD	116 (78.4%)	314 (69.5%)	26 (66.7%)		184 (73.6%)
Type II BD	31 (20.9%)	131 (29.0%)	13 (33.3%)		62 (24.8%)
Schizoaffective, bipolar	1 (0.7%)	7 (1.5%)	0 (0.0%)		4 (1.6%)
History of psychosis, yes *n* (%)	78 (53.4%)	275 (61.7%)	15 (38.5%)		100 (40.7%)
History of suicide attempts, yes *n* (%)	62 (41.9%)	162 (36.2%)	14 (36.8%)		103 (41.5%)
Adult attention deficit disorder, yes *n* (%)	33 (22.8%)	109 (24.4%)	11 (28.2%)		57 (23.2%)
Generalized anxiety disorder, yes *n* (%)	217 (48.5%)	73 (50.7%)	19 (48.7%)		124 (51.7%)
Obsessive compulsive disorder, yes *n* (%)	18 (12.7%)	68 (15.2%)	7 (17.9%)		41 (16.8%)

## Results

A total of 692 subjects (62.7% female, 91.4% White, mean age 43.0 ± 14.0 years) including 176 cases (25.3%) of TEM+ and 516 cases (74.7%) of TEM-with previous exposure to Mito+ and/or Mito− antidepressants were identified. At the time of enrollment into the biobank, TEM+ participants were significantly younger than their TEM− counterpart (40.6 ± 13.8 vs. 43.8 ± 13.9; *p* = 0.009), but there were no significant differences in frequency of BD type I between groups (TEM + 76.1% vs. 70.9%; *p* = 0.31). As shown in Table 2, there were also no large differences in the frequency of history of psychosis or rates of psychiatric comorbidities (Table 2).

**Fig. 2:**
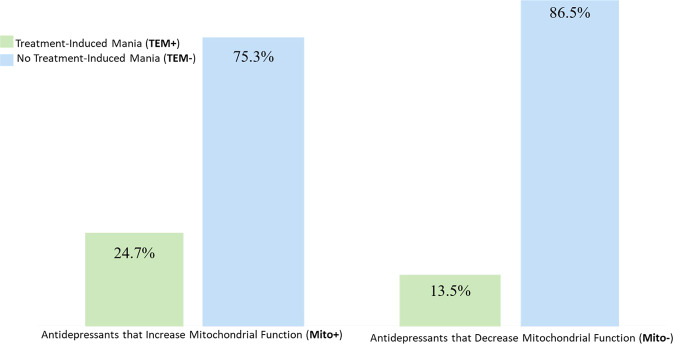
Increased rates of TEM+ with antidepressants that increase mitochondrial function. Rates of treatment-induced mania with antidepressants that increase (Mito+) were two times more frequent than with those that decrease (Mito−) mitochondrial function [TEM+ Mito+ = 24.7%; TEM+ Mito− = 13.5%; OR = 2.21; *p* = 0.000009].

Participants were further classified based on whether the specific AD they had been exposed to increased (Mito+ = 600) or decreased (Mito− = 289) mitochondrial activity; noting that some participants have been exposed to both types of ADs. Adjusting for sex and BD subtype, and after accounting for patient overlap between Mito+ and Mito- groupings, TEM + was more frequent with use of antidepressants that increase mitochondrial activity versus those that decrease it (Mito+ 24.7% vs. Mito− 13.5%; OR = 2.21; *p* = 0.000009) after adjusting for age, sex, and BD type (Fig. 2 and Table 2).

## Discussion

To our knowledge, this is the first study to clinically investigate the mitochondrial energetics profile of specific antidepressants [20] and its association with the adverse drug related event of treatment-emergent mania. When compared to participants exposed to antidepressants that decrease mitochondrial activity, treatment-emergent mania was two times more common in patients exposed to antidepressants that increase mitochondrial energetics. The higher rates of TEM+ observed with Mito+ ADs align with clinical evidence suggesting an increased risk of mood switch with venlafaxine and, to a lesser extent, paroxetine [2, 31–33].

This study has several strengths, most notably a hypothesis driven novel classification of antidepressants beyond conventional drug mechanism of action, and inclusion of a cohort of patients with clinician-defined treatment-emergent mania. One of the main findings from an earlier metanalytic review from our group was the lack of consensus defining the clinical phenotype [26]; the duration of causality of starting antidepressant and subsequent mania for the six studies was up to 52 weeks. The narrower time frame of the phenotype (8 weeks) was a strength of the original study [6, 27] which was used for this current investigation. An additional strength of this study is adjusting for gender and bipolar subtype, which are known risk factors for mood switch [29, 30].

There are a number of study limitations. Due to the retrospective nature of the assessment, age at time of TEM+ occurrence was not obtained, and thus not used as a covariate. Similarly, the study did not control for confounding factors present at the time of study enrollment such as concurrent psychotropic use or comorbid conditions that significantly impact tissue-specific mitochondrial activity (including, but not limited to, diet and BMI, type 2 diabetes, tobacco use, trauma, stress) [34]. These variables should be targeted in future prospective studies assessing TEM and mitochondrial function. Additional limitations include data related to psychotropics used at the time of TEM+ which was not uniformly available. While this is a limitation, previous research has shown the switch rate with antidepressants is greater for BD-I vs BD-II patients, despite greater use of antimanic mood stabilizers [30, 35]; these new clinical data, alongside animal models that suggest an upregulation of mitochondrial activity during acute antidepressant treatment [24] may provide plausible rationale as to mechanism for antidepressants breaking through antimanic mood stabilization. Finally, the classification of antidepressants increasing and decreasing mitochondrial energetics for this clinical study is based on a preclinical systematic review [22]. The preclinical studies were highly variable in study design, with the majority of drugs having mixed results, including the tricyclic imipramine, arguably, after venlafaxine, the antidepressant with the clearest signal for affective switch [36]. While the mixed results category limits strength of the aggregate classification, the data for paroxetine, venlafaxine, nortriptyline, bupropion vs escitalopram and amitriptyline all have preclinical mitochondrial functioning data (state 3/4 respiration, citrate synthase and malate dehydrogenase activity) that are all uniformly positive and negative, respectively. Clearly, future prospective clinical studies on specific antidepressants and mitochondrial activity are encouraged.

As previously mentioned, electron transport chain activity is sensitive to a variety of intrinsic and extrinsic stressors, many of which are common in BD, and to stress mediators (i.e., glucocorticoids) that can lead to a mitochondrial allostatic overload [37]. For instance, early life trauma has been associated with structural and functional mitochondrial changes and impaired energy production; chronic stress is linked with decreased ETC activity, including impaired complex I activity, oxidative stress and mtDNA damage [17, 38]. As an example, it has been hypothesized that suboptimal mitochondrial function in the central nervous system (CNS) can lead to increased likelihood of PTSD through insufficient energy production to cover the increased energy demands of higher CNS glucose metabolism, resulting in increased compensatory complex I activity. Likewise, in the context of PTSD and prolonged stress, release of cytochrome C from the mitochondrial membrane into the cytosol triggers the apoptotic pathway resulting in cell death [39] Within metabolic conditions, obesity and high fat diets reduce mitochondrial number and respiratory capacity, including reduced complex IV and cytochrome C activity, due, in part, to an overload of glucose and fatty acids. The resultant increase in the reduced form of nictotinamide adenine dinucleotide (NADH) production and increased electron availability to the ETC complexes increases reactive oxygen species (ROS) and inflammation [40]. While the overall net effect of obesity is a decrease in mitochondrial energetics, different changes occur through the ETC, including an increase in complexes II and IV and a decrease in complex I and III and ATP synthase [40]. It is worth noting that the specific effects on mitochondria vary not only between diseases but by tissues as well; for instance, in skeletal muscle and adipose tissues, diabetes mellitus and obesity are associated with reduced total activity of the ETC [34, 41]. Contrary to the progressive deleterious effects of metabolic disorders on mitochondrial function, evidence suggests that healthy dietary patterns, including dietary restrictions with and without exercise, indirectly improve mitochondrial capacity by increased expression of genes involved in mitochondrial function [39] Mitochondria are highly sensitive as well to environmental toxicants, such as tobacco smoke, that can alter mitochondrial DNA, oxidant generation and mitochondrial respiration [42]The latter is partially altered by the susceptibility of ETC complexes to inactivation by carbon monoxide leading to a diminished ATP generation. Similar to the impact that healthy lifestyle has on mitochondrial function, cessation of smoking can lead to a restoration of the mitochondrial function and health. Hence the collective relevance of environmental factors that might impact mitochondrial function that, in consequence, might convey increased disease susceptibility in the context of bipolar disorders.

Attempts to clarify the directionality of the complex interrelationship between mitochondrial function and antidepressants must take into consideration the influences exerted by drug combination, duration of treatment and whether there is a cell-type or tissue-specific effect [24]. For example, in preclinical models, the combination of olanzapine/fluoxetine has been shown to increase hippocampal complex I activity both in acute and chronic treatment phases, compared to fluoxetine alone, that increased in acute treatment only [43]. Similarly, Abdel-Razaq et al. (2011) identified, in an in vitro study, that complexes I and IV may be more sensitive to an acute antidepressant-induced inhibition at treatment initiation than other ETC complexes [44]. Lithium is known to increase mitochondrial ETC complex I activity in leukocytes of subjects with bipolar depression, and mitochondrial ETC activity was positively associated with plasma lithium levels [45]. The high rates of polypharmacy in BD may interfere with the measurement of ETC activity, adding a layer of complexity to the assessment of the interrelationship of mitochondrial function and psychotropics [45].

Understanding the primary pathophysiology of ETC dysfunction in BD (i.e., disease risk) may help guide pharmacogenomic studies. A systematic review of 10 ETC microarray gene expression studies in BD would suggest a main driver of ETC dysfunction is in complex I, with reduced gene expression of *NDUFV1*, *NDUFS1*, *NDUFS8*, and *NDUFS7*. Importantly, NDUFS7 directly couples electron transfer between the iron sulfur cluster and ubiquinone, a critical exchange of electrons for cellular energy production [12, 46]. Assessment of the ETC complex I, the entry enzyme of oxidative phosphorylation, and complex IV, the final enzyme with a rate-limiting role in the cellular respiration, may serve as a proxy of mitochondrial bioenergetics of the brain in psychiatric disorders, as they are known to be impacted by psychotropics [47, 48]. This is further supported by clinical data on upregulation of complex I subunits during mania compared to depressive episodes and healthy controls, suggesting mitochondrial complex activation [48, 49]. Our group previously identified, through mtDNA sequence data of BD-1 patients (*n* = 224), a higher risk of psychosis with U haplogroup, as well as a variation in ND4 gene, implicated in ETC energy regulation [50]. Additionally, increased ceramide concentrations, involved in mitochondria-mediated apoptosis and associated with decreased activity of complexes I, IV and V, have been demonstrated in individuals with BD [51–53] and are likely aggravated by certain antidepressant medications (i.e., fluoxetine, fluvoxamine, paroxetine, escitalopram and amitriptyline) [54].

In conclusion, our study provides early evidence that support the hypothesis of an amplified response in mitochondrial energetics of select antidepressants that drive, in part, the pathophysiology of treatment-emergent mania. In addition, these data suggest categorizing antidepressants based on mitochondrial energetics, and not solely monoaminergic conventional drug mechanism of action (SSRI, SNRI, TCA, MAOI), may be of value and warrant further consideration for future larger clinical and pharmacogenomic studies. Recognizing pharmacogenomic investigation of drug response may extend or overlap to genomics of disease risk, future clinical studies should investigate potential interactions between mitochondrial mechanisms of disease risk that may predispose to antidepressant associated treatment-emergent mania and may have a clear impact on the management and treatment of patients with bipolar disorder.
